# dgfr: an R package to assess sequence diversity of gene families

**DOI:** 10.1186/s12859-024-05826-2

**Published:** 2024-06-06

**Authors:** Laila Viana Almeida, João Luís Reis-Cunha, Daniella C. Bartholomeu

**Affiliations:** 1 Departamento de Parasitologia, Instituto de Ciências Biológicas, Universidade Federal de Minas Gerais, Belo Horizonte, Brazil; 2 Department of Biology, York Biomedical Research Institute, University of York, York, UK

**Keywords:** Sequence diversity, Gene families, Clustering

## Abstract

**Background:**

Gene families are groups of homologous genes that often have similar biological functions. These families are formed by gene duplication events throughout evolution, resulting in multiple copies of an ancestral gene. Over time, these copies can acquire mutations and structural variations, resulting in members that may vary in size, motif ordering and sequence. Multigene families have been described in a broad range of organisms, from single-celled bacteria to complex multicellular organisms, and have been linked to an array of phenomena, such as host–pathogen interactions, immune evasion and embryonic development. Despite the importance of gene families, few approaches have been developed for estimating and graphically visualizing their diversity patterns and expression profiles in genome-wide studies.

**Results:**

Here, we introduce an R package named dgfr, which estimates and enables the visualization of sequence divergence within gene families, as well as the visualization of secondary data such as gene expression. The package takes as input a multi-fasta file containing the coding sequences (CDS) or amino acid sequences from a multigene family, performs a pairwise alignment among all sequences, and estimates their distance, which is subjected to dimension reduction, optimal cluster determination, and gene assignment to each cluster. The result is a dataset that allows for the visualization of sequence divergence and expression within the gene family, an approximation of the number of clusters present in the family.

**Conclusions:**

*dgfr* provides a way to estimate and study the diversity of gene families, as well as visualize the dispersion and secondary profile of the sequences. The *dgfr* package is available at https://github.com/lailaviana/dgfr under the GPL-3 license.

**Supplementary Information:**

The online version contains supplementary material available at 10.1186/s12859-024-05826-2.

## Background

Gene families consist of groups of genes that share a common evolutionary origin, often exhibiting similar biological functions. These families emerge through gene duplication events over the course of evolution, resulting in multiple copies of an ancestral gene. Over time, these copies can accumulate mutations and undergo sequence divergences, leading to variations in size, ordering of motifs that can alter function and structure among family members. These gene families manifest across a diverse range of organisms, spanning from bacteria to humans and might be organized in the genome in clusters, repeated in tandem, or dispersed across different chromosomes [1]. In some protozoan parasites, these gene families may encode proteins located at the cell surface, where they play a pivotal role in host-parasite interactions such as cell invasion and immune evasion [2]. Numerous studies undertake phylogenetic analyses of these families, however some crucial steps for phylogeny, such as multiple alignments may be challenging due to differences in size and motif ordering that may occur across members of polymorphic gene families. To address this, we have developed an R package that generates a distance matrix from the pairwise comparison of each gene sequence in a multigene family, followed by dimension reduction and the determination of the optimal number of clusters within the family and generates graphic representations.

## Implementation

**Fig. 1:**
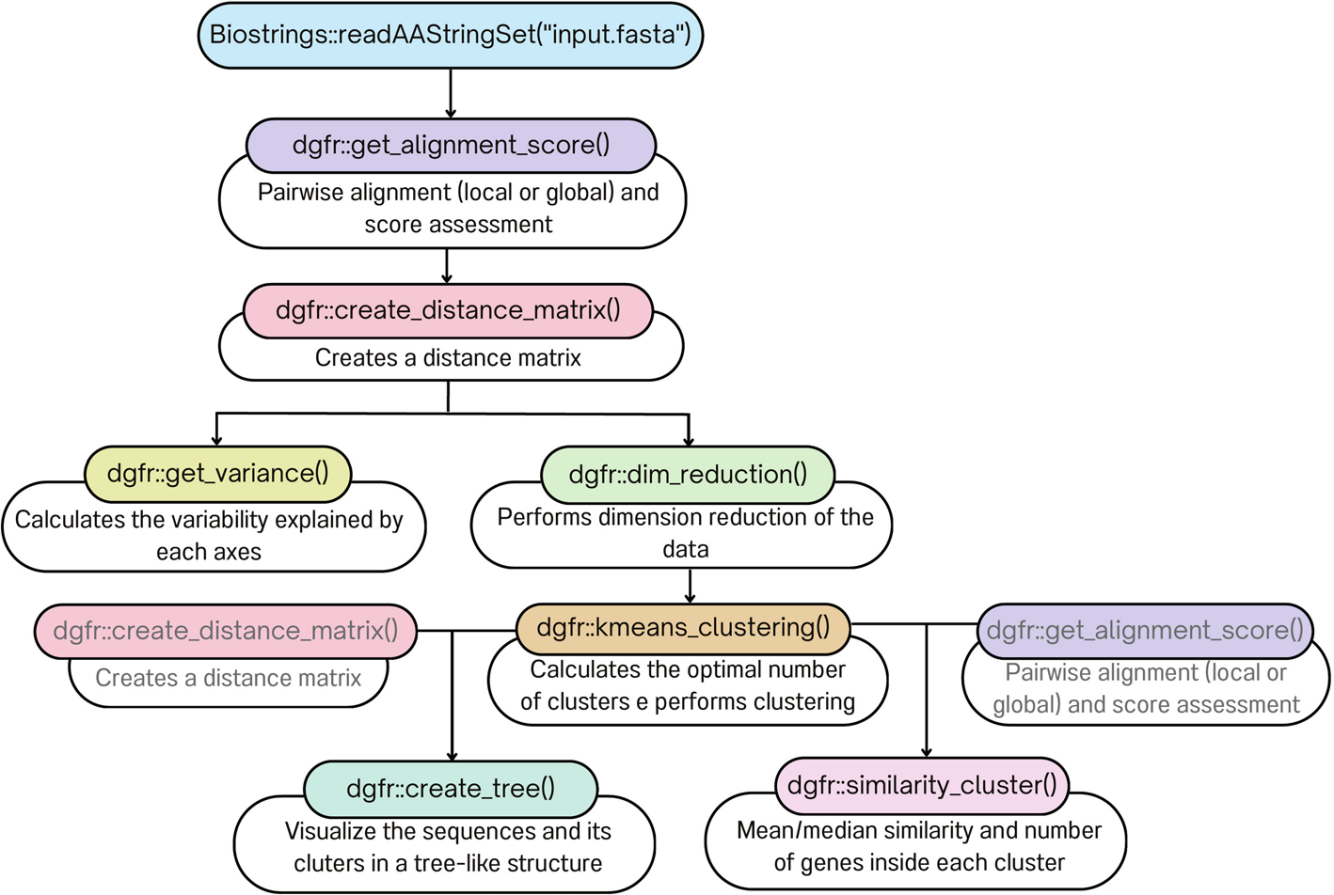
Flowchart showing the input file and the dgfr package's operations

The 'dgfr' package requires 'BiocManager' from CRAN and 'Biostrings' from 'BiocManager' to be installed and loaded into the R environment before installation. Please refer to the 'dgfr' GitHub page for information on installing these dependencies. The functions 'readAAStringSet()' or 'readDNAStringSet()' from the 'Biostrings' package are required to read the protein or the nucleotide multi-fasta file provided by the user, respectively. A small dataset is provided to allow a rapid test of the package. To use this test fasta file, the user must call ‘data(fasta)’ as input. This package comprises seven distinct functions (Fig. 1).

‘**get_alignment_score**’: is responsible for reading the fasta object, previously read by Biostrings functions readAAStringSet() or readDNAStringSet(), performing the pairwise alignment and calculating the normalized score for every possible combination between the sequences. This score is normalized by sequence length as proposed by Bleakly and Yamanashi [3]. This function has three other mandatory arguments. The first indicates whether the user is providing a nucleotide sequence or a protein sequence, which guide the substitution matrix used. The blast matrix is utilized for nucleotide sequences, while the BLOSUM62 matrix is used for protein sequences. The second indicates if the user wants to use a local or global alignment. The user is then provided with a dataframe containing similarity scores ranging from 0 to 1 for every pair of sequences. The third argument allows the user to specify the number of cores the process will use, which is set to 3 by default. The output generated by this function can be used as input in several dgfr functions, which are further described below.

‘**create_distance_matrix**’: uses as input the output of the ‘get_alignment_score’ and uses the dist() function from the stats package to compute the Euclidean distance between each pair of sequences. It generates a distance matrix as output.

‘**get_variance**’: uses the function get_eig() from factoextra package to compute how much of the data variance that is explained by each axis of the distance matrix and produces a dataframe that encompasses both the axis information and the corresponding percentage of variance. This information can be subsequently utilized to construct the axis titles of the PCA (Principal Component Analysis) graph.

‘**dim_reduction**’: takes the distance matrix produced by the ‘create_distance_matrix’ function as its input. Dimension reduction is performed using the cmdscale() function from stats package on the matrix, resulting in its transformation into a two-dimensional representation. A dataframe containing the sequence names and their respective positions on axis_1 and axis_2 is produced by the function.

‘**kmeans_clustering**’: accepts three arguments, of which only the first is mandatory. This argument will provide the output yielded by the ‘dim_reduction’ function. The other two arguments denote the minimum and maximum quantity of clusters to be assessed within the dataset. If these values are not specified, the function will assess the range of two to twenty clusters. Upon determining the optimal cluster count with the function NbClust from the package NbClust, the next task of the function is to perform sequence clustering using the k-means algorithm. The outcome of this process contains the sequence names, their respective positions on axis_1 and axis_2, and the assigned cluster for each sequence.

‘**similarity_cluster**’: calculates the mean and median similarity among each cluster. This function accepts the output of ‘get_alignment_score’, ‘kmeans_clustering’ as inputs. Additionally, it requires the selection of one of the two output modes, one of which is referred to as “table” generating a data frame containing the cluster, the mean and median similarity, and the number of genes within each cluster. “plot” is an alternative option that generates a ggplot2 graph representing the extent of similarity within each cluster.

**‘create_tree’:** this function allows you to visualize the sequences and its cluster in a tree-like structure using the ggtree package. You must provide the kmeans_clustering function output and the create_distance_matrix output. The output will be a plot containing the sequences and its cluster.

## Results

Once the data frame from the ‘kmeans_clustering’ is generated, that data can be visualized using the ‘ggplot2’ package or other similar tools and packages. In addition, it is possible to incorporate further information, such as RNAseq data, into the visualization. By leveraging these capabilities, the plots created by dgfr provide insights into the relationships among sequences, clusters, and additional data, enhancing the interpretability and depth of the analysis.

As a case study, we have chosen a variable gene family named trans-sialidase that is expressed on the surface of the *Trypanosoma cruzi* parasite*,* the etiological agent of Chagas disease. This parasite infects a great range of mammals, including humans, and is transmitted by Triatominae vectors [4]. The trans-sialidase family has 616 identified proteins in the YC6 strain [5]. These proteins play a pivotal role in the parasite's survival, and some genes of this family are responsible for transferring sialid acid from the host to the mucin residues located on the parasite’s surface [6, 7]. Other functions related to the trans-sialidases include their capacity to bind to certain cell types [2].

**Fig. 2:**
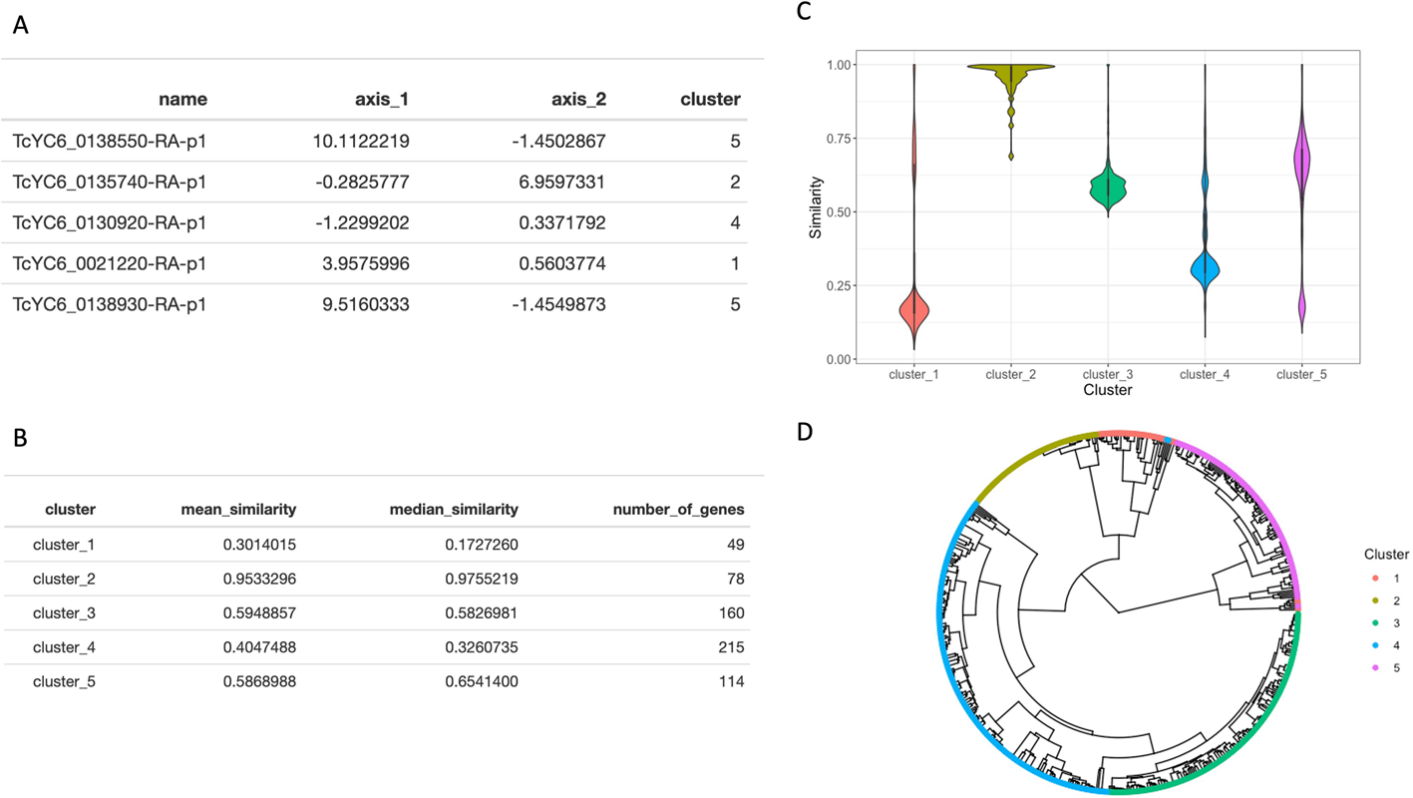
Main outputs of the dgfr package. **A** The result of kmeans_clustering, which displays the genes' name, its position in axis_1 and axis_2 and its assigned cluster. **B** The similarity_cluster table's output, which depicts the cluster, the mean and median similarity within each cluster and the number of genes per cluster. **C** Plot from similarity_cluster function showing the degree of similarity within each cluster. **D** Plot from create_tree function, showing the genes and its cluster in a tree structure

To visualize the diversity of the trans-sialidase gene family, the sequences of protein-coding genes were imported into R and then to the dgfr package using Biostrings::readAAStringSet() function. The main outputs of the described package are two tables and two plots. The first table, which contains the name of the gene, its position on axis 1 and 2, and the cluster to which it belongs, is the result of the kmeans_clustering function. The cluster, the mean and median similarity within the cluster, and the number of genes in each cluster are displayed in the second table, which is the output of similarity_cluster. The first plot is the output of the similarity_cluster function, that illustrates the extent of sequence similarity within each cluster and the second plot is the output of the create_tree function, showing the sequences and clusters inside a tree structure (Fig. 2).

**Fig. 3:**
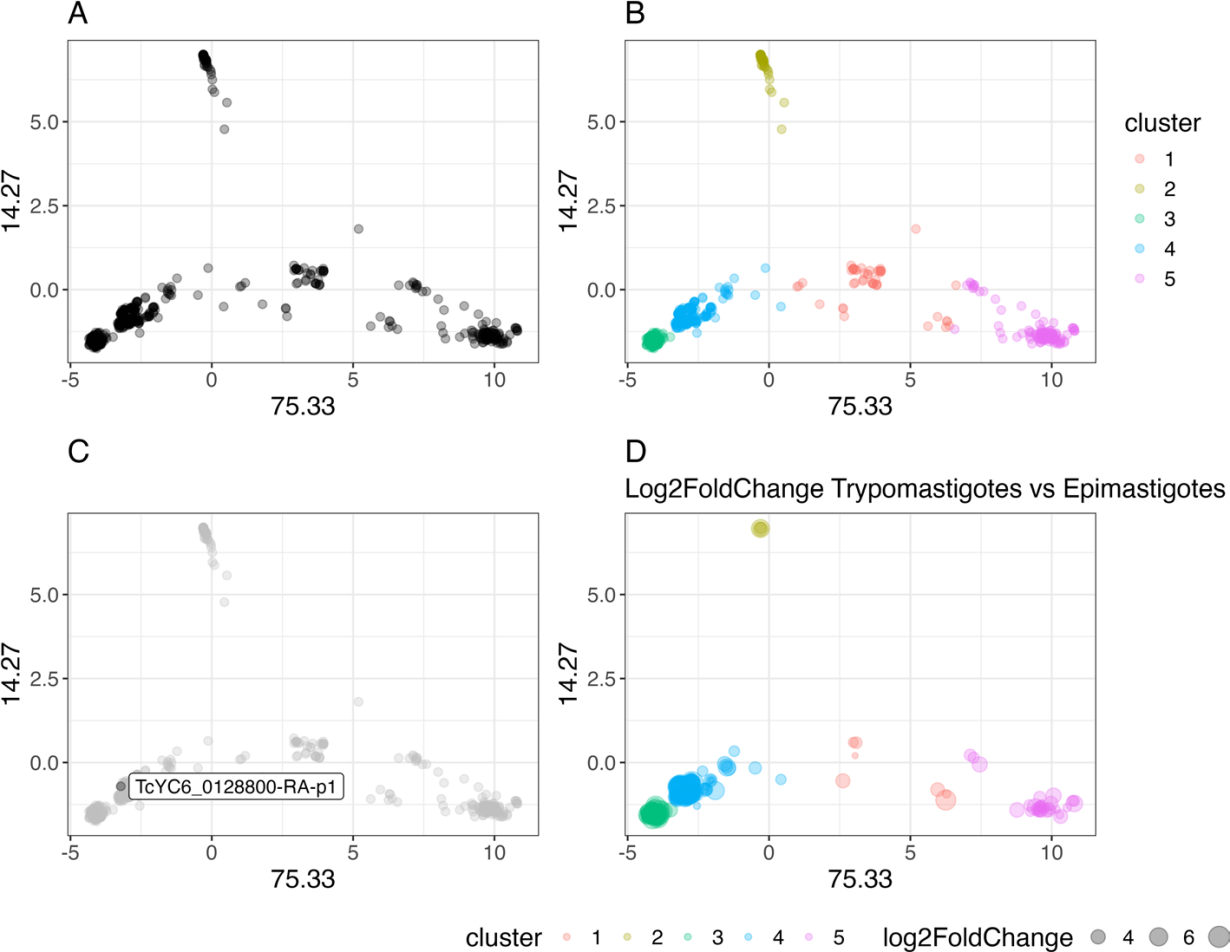
Trans-sialidase gene family dispersion pattern. **A** Principal Component Analysis (PCA) showing the dispersion pattern of the gene family. **B** PCA displaying the total number of identified clusters within the family. **C** PCA highlighting a particular protein within a given protein family. **D** PCA is employed to identify and emphasize the genes that exhibit differential expression between trypomastigote and epimastigote forms of the parasite within the family. The sizes of the dots, which correspond to the log2FoldChange, are proportional to the expression levels of the genes

Numerous other visualizations can be produced in addition to the output tables and plots generated by the dgfr package's functions. Here we present four distinct approaches to visualize a multigene family. First, the dispersion of the family can be assessed; second, the optimal number of clusters within the family can be visualized; third, a particular sequence within the family can be highlighted; and finally, RNA-seq data can be incorporated to illustrate the expression levels of each sequence under different conditions (Fig. 3). The code used to generate the Fig. 3 is available in the Additional File 1 and the RNA-seq analysis is described in Additional File 2.

## Conclusions

The dgfr R package allows the examination and graphical representation of the sequence diversity of complex gene families. This includes the ability to detect clusters and integrate expression data. It is an automated tool that boasts a user-friendly interface and operates with minimal complexity, only requiring the fasta sequence of members of a given gene family as an input file. The dgfr package is available at https://github.com/lailaviana/dgfr.

## Availability and requirements

- Project name: *dgfr*
- Project home page: https://github.com/lailaviana/dgfr
- Operating system(s): Platform independent
- Programming language: R
- Other requirements: None
- License: GPL-3.0 license
- Any restrictions to use by non-academics: None

### Supplementary Information

Additional file1.

Additional file2. 

## Data Availability

The *dgfr* package and the datasets analysed during the current study are available on GitHub (https://github.com/lailaviana/dgfr).
