# Supplementary material for: dgfr: an R package to assess sequence diversity of gene families
Source: BMC Bioinformatics. 2024 Jun 6;25:207. doi: 10.1186/s12859-024-05826-2 (PMC11155016; doi:10.1186/s12859-024-05826-2)
Supplement: Supplementary file 1 — Additional file1. [file 12859_2024_5826_MOESM1_ESM.docx]

**Additional File 1.** Code used to generate the Figure 3.

#installing and loading packages ----------

#devtools::install_github("lailaviana/dgfr")

library(tidyverse)

library(dgfr)

library(Biostrings)

library(patchwork)

#reading fasta file ------------

ts_fasta <- readAAStringSet("ts_paper/ts_prot.fasta")

#running dgfr functions -----------

score_ts <- dgfr::get_alignment_score(ts_fasta,

type = "prot",

alignment_method = "global")

ts_dist_matrix <- dgfr::create_distance_matrix(score_ts)

ts_variance <- dgfr::get_variance(ts_dist_matrix)

ts_dim_red <- dgfr::dim_reduction(ts_dist_matrix)

ts_kmeans <- dgfr::kmeans_clustering(ts_dim_red)

ts_kmeans |> slice_sample(n = 6) %>% gt::gt()

sim_cluster_table <- dgfr::similarity_cluster(score_ts,

ts_kmeans,

"table")

sim_cluster_table %>% gt::gt()

#creating the figures ----------

#figA ------

figA <- ts_kmeans |>

ggplot(aes(x = axis_1, y = axis_2)) +

geom_point(size = 2, alpha = 0.3) +

theme_bw() +

labs(x = round(ts_variance$`1`, 2),

y = round(ts_variance$`2`, 2),

title = "A") +

theme(text = element_text(size = 15))

#figB ------

figB <- ts_kmeans |>

mutate(cluster = as.character(cluster)) |>

ggplot(aes(x = axis_1, y = axis_2, color = cluster)) +

geom_point(size = 2, alpha = 0.3) +

theme_bw() +

labs(x = round(ts_variance$`1`, 2),

y = round(ts_variance$`2`, 2),

title = "B") +

theme(text = element_text(size = 15))

#figC ---------

highlight_gene <- sample(ts_kmeans$name, 1)

figC <- ts_kmeans |>

ggplot(aes(x = axis_1, y = axis_2)) +

geom_point(size = 2, alpha = 0.3) +

theme_bw() +

labs(x = round(ts_variance$`1`, 2),

y = round(ts_variance$`2`, 2),

title = "C") +

theme(text = element_text(size = 15)) +

gghighlight::gghighlight(name == highlight_gene, label_key = name)

#figD -----------

kmeans_res <- read_csv("ts_paper/kmeans_res.csv")

rnaseq_mucin <- read_tsv("ts_paper/rnaseq_trypo_mucin.tsv")

upregulated_mucin_trypo <- rnaseq_mucin |>

mutate(gene = paste0(gene, "-RA-p1"))

kmeans_ts <- kmeans_res |>

left_join(upregulated_mucin_trypo, join_by(name == gene))

figD <- kmeans_ts |>

mutate(cluster = as.character(cluster)) |>

ggplot(aes(x = axis_1,

y = axis_2,

size = log2FoldChange,

color = cluster)) +

geom_point(

alpha = 0.3) +

theme_bw() +

labs(x = round(ts_variance$`1`, 2),

y = round(ts_variance$`2`, 2),

title = "D",

subtitle = "Log2FoldChange Trypomastigotes vs Epimastigotes") +

theme(text = element_text(size = 15),

legend.position = "bottom")

#joining figures ----------

(figA + figB) / (figC + figD)

ggsave(filename = "ts_paper/figure_ts.png",

plot = last_plot(),

device = "png",

width = 10, height = 8, dpi = 600)
