# Supplementary material for: dgfr: an R package to assess sequence diversity of gene families
Source: BMC Bioinformatics. 2024 Jun 6;25:207. doi: 10.1186/s12859-024-05826-2 (PMC11155016; doi:10.1186/s12859-024-05826-2)
Supplement: Supplementary file 2 — Additional file2. [file 12859_2024_5826_MOESM2_ESM.docx]

**Additional file 2.** RNA-seq analysis depicted in Figure 3.

The RNA-seq data presented here were derived from a previously conducted investigation and were made publicly available through the Sequence Read Archive (SRA) under the accession PRJNA251583. Briefly, the reads obtained from the SRA were subjected to quality assessment using the fastqC software (http://www.bioinformatics.babraham.ac.uk/projects/fastqc) and were then filtered using the trimmomatic software [1]. The filtered reads were mapped against the YC6 genome version 64 obtained from TriTrypDB (https://tritrypdb.org/tritrypdb) using the STAR program [2]. The read counts were evaluated using the featureCounts software [3]. The differential expression analyses were conducted using DESEQ2 [4] and genes were considered upregulated when the p adjusted values were < 0.05 and the log2foldchange were higher than 2.

**References**

1. Bolger AM, Lohse M, Usadel B. Trimmomatic: a flexible trimmer for Illumina sequence data. Bioinformatics. 2014;30:2114–20.

2. Dobin A, Davis CA, Schlesinger F, Drenkow J, Zaleski C, Jha S, et al. STAR: ultrafast universal RNA-seq aligner. Bioinforma Oxf Engl. 2013;29:15–21.

3. Liao Y, Smyth GK, Shi W. featureCounts: an efficient general purpose program for assigning sequence reads to genomic features. Bioinforma Oxf Engl. 2014;30:923–30.

4. Love MI, Huber W, Anders S. Moderated estimation of fold change and dispersion for RNA-seq data with DESeq2. Genome Biol. 2014;15:550.
